# Patient and Provider Cocreation of Mobile Texting Apps to Support Behavioral Health: Usability Study

**DOI:** 10.2196/12655

**Published:** 2020-07-29

**Authors:** Armen C Arevian, Jennifer O'Hora, James Rosser, Joseph D Mango, David J Miklowitz, Kenneth B Wells

**Affiliations:** 1 Jane and Terry Semel Institute for Neuroscience and Human Behavior University of California Los Angeles Los Angeles, CA United States

**Keywords:** mobile health, community-based participatory research, app development, technology platforms, personalized medicine, behavioral health, mobile phone

## Abstract

**Background:**

Mobile technologies hold potential for improving the quality of care and engagement of patients. However, there are considerable challenges in ensuring that technologies are relevant, useful, and engaging. While end users such as patients and providers are increasingly involved in the design of health technologies, there are limited examples of their involvement in directly creating technologies for their personal use.

**Objective:**

We aim to evaluate the feasibility and acceptability of patients and providers creating mobile texting apps to support treatment goals.

**Methods:**

In an 11-month usability study, we enrolled 4 providers and 28 patients in an intensive outpatient program for obsessive-compulsive disorder. Patients and providers created their own mobile texting apps using a visual app development platform. A subsample of 10 patients and 4 providers completed a usability measure.

**Results:**

Participants created a total of 360 unique mobile text messages (1787 total messages sent). There were 4 types of messages identified, including personalized reminders, clinical exposures, interactive prompts, and encouraging/informational messages. A total of 9 out of 10 (90%) patients agreed that the messages were relevant to their recovery, and 8 out of 10 (80%) agreed that the messages were effective at helping complete treatment plans.

**Conclusions:**

Enabling patients and providers to cocreate apps for their own use by using a visual application platform is feasible and holds potential for increasing the relevance, sustainability, and effectiveness of digital health technologies.

## Introduction

Digital technologies such as mobile apps are increasingly used to improve the quality of care and engagement of patients [1], especially for chronic disease management, in which engagement of individuals over time is challenging [2]. There are considerable barriers in ensuring that technologies are relevant, useful, and lead to sustained use at the patient, provider, and institutional levels [3,4]. Involving broader stakeholders, including patients and providers, is increasingly seen as critical in addressing these challenges. However, their involvement in the development process is typically limited to the design of apps, often providing feedback on the content, functionality, and visual appearance. Translating these designs into functioning apps is often undertaken separately by computer programmers, resulting in the removal of the end users from this aspect of development, as well as additional cost, complexity, and time. There have not been previous reports of a systematic approach to engaging patients and providers as the direct creators of mobile health apps that they use to support their health needs [5].

The technology adoption model identifies several intrinsic and extrinsic factors related to technology use, including perceived ease of use and relevance [6]. Both of these factors may be negatively impacted when apps are not created for specific individuals or local groups but rather made to address broader diseases (eg, diabetes management), conditions (eg, weight loss, stress reduction), or approaches (eg, cognitive behavioral therapy). At the same time, direct engagement by patients has been recognized as a key determinant of health behavior change, better health outcomes, and satisfaction [7].

**Figure 1:**
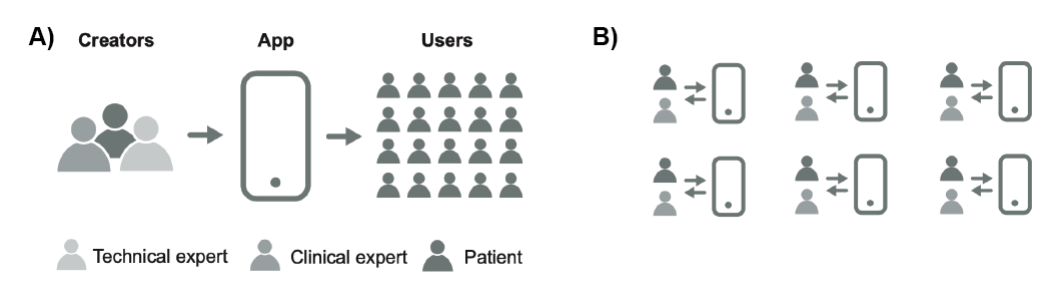
Comparison of two approaches to the participatory technology development process for mobile apps. A) Stakeholders cocreate an app for a group of end users. B) Patients and providers cocreate apps to support themselves.

Participatory approaches, including participatory design and community-partnered participatory research [8,9], were created to involve stakeholders such as patients, providers, and community leaders, and are increasingly used in a variety of settings, including those involving digital health [5]. However, the translation of designs to app creation is often undertaken separately by technical individuals such as computer programmers. In addition, existing participatory methods often aim to create an app for a population whose users were not involved in the process. This is different than a systematic approach to involving individuals in the creation of an app for their personal use, as seen in Figure 1.

A major assumption of the present study is that when individuals are involved in creating the apps that they will personally use, their engagement and the perceived usefulness of the apps will be enhanced. We created the Chorus application platform (Chorus Innovations Inc) [10] to facilitate this process of participatory technology development, in which stakeholders can be directly involved in both designing and creating mobile apps. An application platform provides modules and functions that can be reused to create specific applications to reduce development time, lower cost, and improve scalability [11]. Furthermore, Chorus is a no-code application platform that uses a visual interface to configure apps without the need for computer programming, with the objective of supporting a broad population of stakeholders as app creators [12]. Chorus has been used to create mobile web, text messaging, and interactive voice apps for more than 50 research and clinical projects, including ongoing studies of automated text messaging interventions for asthma [13] and healthy lifestyle [14].

Our group previously used the Chorus platform to facilitate a participatory development process to cocreate a mobile texting app to support resiliency (B-RESILIENT) [15]. We conducted a series of partnered workgroups with stakeholders, including patients, community leaders, and academic researchers. In these workgroups, key needs of the community, selected and adapted content to include in the app, and privacy and other concerns related to trust of the technology were discussed. The workgroups then created an app that addressed these needs.

It remains an open question to what extent it is feasible for individual patients and providers to directly create personal mobile apps for their own use in real-world clinical settings. The goal of this pilot study was to evaluate the usability of the Chorus platform and the feasibility of a participatory development process in the creation of mobile texting apps by patients and providers, as well as to describe the kinds of mobile texting apps created by these participants. The study was conducted with patients in an intensive outpatient treatment program and their providers.

## Methods

### Setting and Participants

This study was conducted in the Obsessive-Compulsive Disorder (OCD) Intensive Outpatient Program (IOP) of University of California Los Angeles (UCLA) Health. Patients with OCD attended the IOP program 3 to 5 weekdays per week. The IOP uses cognitive behavioral therapy (CBT) techniques, including exposure to situations and stimuli that trigger anxiety symptoms, known as Exposure and Response Prevention (ERP) [16]. The core of ERP is intentional and planned exposure to stimuli that trigger anxiety responses, with progressively increasing levels of stimulus threat as treatment progresses.

Key clinical challenges were identified in a workgroup meeting with IOP staff and research personnel at the beginning of the study. The IOP staff identified low patient satisfaction with support after clinic hours and the difficulty of completing exposure tasks outside of the clinic setting, a common barrier in OCD treatment [17].

The Chorus platform was implemented by the IOP as part of clinical care rather than as part of a research study. Patients who enrolled in the IOP were consented by clinic staff for use of text messaging as part of their care. Those that agreed were then consented by research staff to participate in this study. The scope of the research study was to evaluate the clinic’s implementation and use of the Chorus platform by analyzing text messages, administering usability measures, and conducting workgroups. All research methods were approved by the UCLA Institutional Review Board.

Inclusion criteria for study participation included being an active patient of the IOP clinic, having a mobile phone (smartphone not required), and being willing to receive text messages. Patients used their personal mobile phones and interacted with their app through text messaging. There was no app to download. There were no exclusion criteria. We chose these broad criteria to reflect the typical population from this clinic. Technical skills such as computer programming were not a requirement for participation.

During the pilot period, there were 4 PhD psychology providers who participated in the study. The number of patients active in the clinic at any given time varied between 6 and 8 patients. Patients participated in the study for as long as they were enrolled in the IOP (average stay of 6-8 weeks). No participants disenrolled early from the study.

### Participatory Technology Development

**Figure 2:**
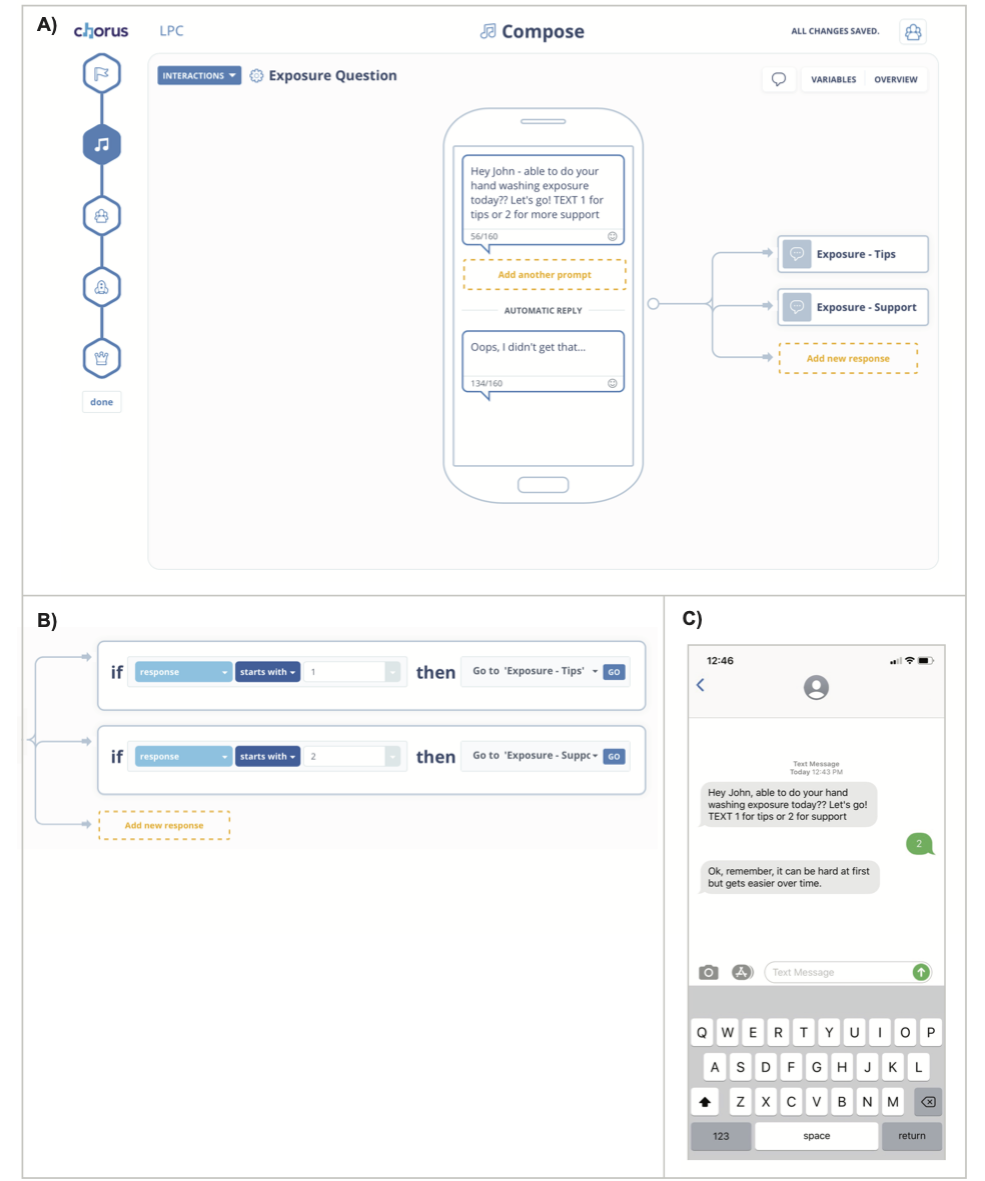
Visually creating apps with Chorus. A) Creation of text messaging content visually with a simulated phone. B) Visual interface to configure the logic that guides subsequent messages to send based on user’s response. C) Screenshot of an example mobile texting interaction as seen by patients.

The clinic implemented a process of participatory technology development (Figure 1) to facilitate patients and providers to cocreate personal mobile text messaging apps using the Chorus platform created by Arevian et al [15]. Providers were given log-in access to the platform and used the visual web interface with their patients to cocreate the texting apps, as seen in Figure 2. This included specifying the content of messages, logic to handle responses from the patient if needed, and scheduled times to send the messages to the patient from the platform. The content and functionality of the apps were entirely driven by the patient and provider. The messaging did not facilitate direct communication between patients and providers. Instead, the app was configured to automatically send the messages to the patient at a later time. If the patient replied to the message, they would receive an automated preconfigured (by the patient and provider) response from the app.

Initial training for clinic psychotherapists to use Chorus was a single 1-hour session. One therapist requested an additional training session. Therapists were not provided specific guidance on the types of apps to create other than to address the clinical needs of their patients. Therapists reviewed the app’s functionality with patients as needed as part of the cocreation process. The Chorus platform was provided through the Innovation Lab at the Jane and Terry Semel Institute for Neuroscience and Human Behavior at UCLA.

### Measures and Analysis

Demographic details (age, sex, race) and clinical outcomes were extracted from patients’ medical records. We report the Yale-Brown Obsessive-Compulsive Scale (Y-BOCS) score to describe the clinical severity of this population. Y-BOCS is a measure of OCD symptom severity and was assessed by study staff on admission as part of routine care [18].

Feasibility and acceptability were evaluated through a written questionnaire that study staff administered to patients. The measure included items of perceived ease of use, perceived usefulness, self-efficacy, and patient-centeredness (see Multimedia Appendix 1) [6,19]. Participants indicated their agreement with statements in the survey using a Likert scale ranging from 1 to 7, where 1 indicated they strongly disagreed, 4 indicated they were neutral, and 7 indicated they strongly agreed with the statement. The survey also included 2 open-ended prompts to list the most positive and negative aspects of their apps, the platform, or the process of participatory development. The survey was administered to 14 participants (10 patients and 4 therapists) during a 2-month evaluation period of the pilot.

We conducted a total of 7 workgroups with patients and 7 with providers to discuss experiences related to the app development and participatory process. Each workgroup typically had 2 to 7 participants and lasted 30 minutes to 1 hour. Workgroup sessions were audiorecorded and reviewed by study staff. Qualitative analyses were conducted by reviewing transcripts (for workgroups) and message logs (for app content) to group phrases or messages together and identify themes [20]. Themes were cross-checked by 2 study staff and reviewed with clinic staff for validity. Representative quotes and messages are tabulated in the results below. App use metrics were extracted from the Chorus activity logs for all participants.

Retention rates for the use of the mobile texting apps were calculated using the date of the last message sent or received by the patient from the Chorus logs and comparing this to the date of termination from the clinical program.

## Results

We conducted a rolling enrollment over an 11-month period between July 2015 and May 2016. A total of 28 patients and 4 providers were enrolled in the study during this period. The providers were all PhD-level psychotherapists specializing in the treatment of anxiety disorders. Of the 28 patients, 11 were female (39%). Mean age was 33 years (SD 14.2; range 18-69). Patients were mostly white (26/28, 93%) and had severe OCD symptom burden on admission (Y-BOCS mean score of 38 out of 40, indicating extreme level of symptoms).

A total of 1787 messages were sent to patients during the evaluation period (360 unique messages) and 80 responses were received from patients. Patients received an average of 51 messages (SD 55). All patients responded at least once to a text message. The number of messages sent did not vary by day of the week. Most text messages were sent after clinic hours (between 2 PM and 10 PM).

We identified 4 basic types of messages created, including personalized reminders (ie, prompting of a clinical goal to be achieved at home), messages serving as clinical exposures (ie, intended to evoke feared stimuli), interactive prompts (messages that requested a response from the patient), and encouraging or informational messages (Textbox 1). A common theme of the messages was to use personalization and humor to create messages related to treatment engagement.

**Content of mobile texting apps.**

**Interactive**

- “Were you able to sit with the anxiety and contamination without washing today? Press 1 for yes and 2 for no”
- “Hey there {patient name}, did you meet your goal today? TEXT 1 for yes or 2 for no”
- “Don't forget to take the Namenda tonight. Press 1 if you took the medication”
- “Hey, did you go to the store today?! TEXT: 1 for yes, 2 for no”

**Personalized reminders**

- “Hi {patient name}, No more showering. Life is too short. Lean into the grubbiness. You can do this”
- “It's 10pm, time to give up your devices. They turn into pumpkins!”
- “Good morning {patient name}, here is your sleep diary reminder! {link to diary website}”
- “Ready, set, go - shower in under 12. Otherwise get ready to shower at [University of California Los Angeles] in 6 minutes”
- “Leave those faucets alone {patient name}. You don't need them”
- “C'mon tin ribs, get to the gym and feel the burn!”

**Message as the exposure (exposure type)**

- “Remember, Typhoid {patient name}, No handwashing. You are a walking petri dish!” (contamination)
- “Showering once a day means you have some pretty gross stuff on you” (contamination)
- “gr8t job this week - don't forget two exercise this weekend :)” (spelling)
- “{expletive}” (unwanted thoughts)

**Encouraging/informational**

- “Gold star Mr. Jelly Legs. Be proud of yourself”
- “Remember to try to go as long as possible without washing, even if you feel contaminated. You are stronger than your [obsessive-compulsive disorder]!”
- “Very good padawan”
- “Exercise combats health conditions and diseases. It can also improve mood”
- “That's awesome!! Great Job!”

Results from the usability survey for patients and providers are presented in Table 1. Individuals responded to how much they agreed with the statements listed in Table 1 using a Likert scale, where 1=strongly disagree, 4=neutral, and 7=strongly agree. Representative open-ended responses for positive and negative features of the system are included in Textbox 2. Regarding the use of the Chorus platform, 11 out of 14 (79%) participants (10 patients and 4 providers) agreed that Chorus was simple to use, 12 out of 14 (86%) felt comfortable using Chorus, 13 out of 14 (93%) felt comfortable using the text messaging app, and 11 out of 14 (79%) felt they (or their patients) were more engaged as a result. All providers (4/4, 100%) and most patients (8/10, 80%) agreed or responded neutrally to the statement “I can effectively develop messages with my provider (patient) using this messaging application.” Of the 10 patients surveyed, 9 (90%) agreed that the messages were relevant to them and their recovery, and 8 (80%) agreed that the messages were effective in helping them complete treatment exercises at home.

**Figure 3:**
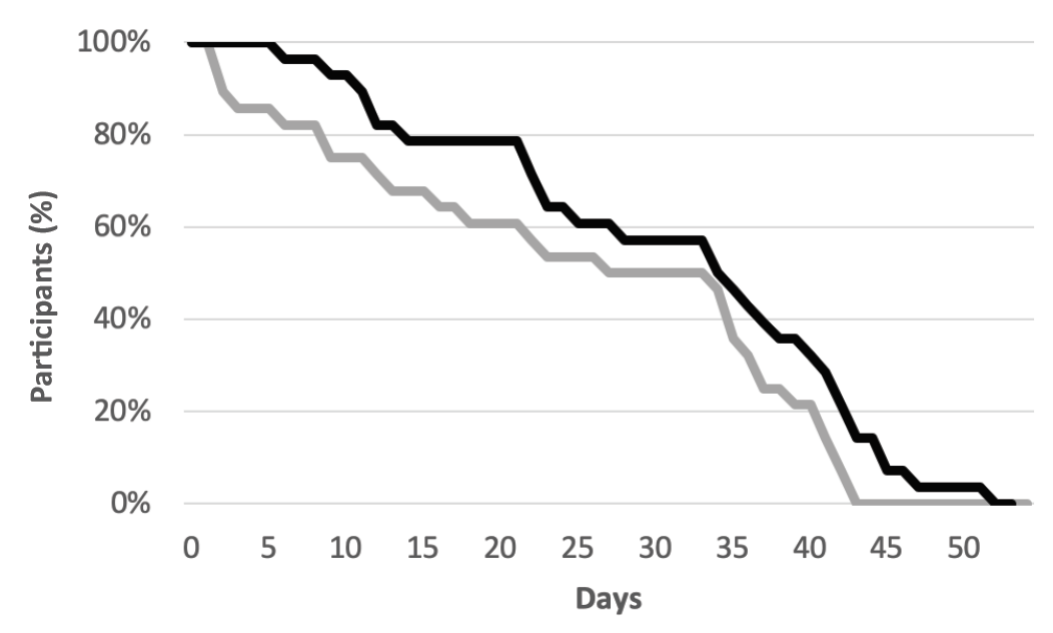
Patient engagement. Percentage of participants enrolled in the Intensive Outpatient Program clinic (black line) compared with the percentage of participants actively using their mobile app (grey line) by number of days in the program.

Overall, engagement with the cocreated apps was sustained over the time the patient was engaged in the clinic (Figure 3). The average duration of mobile app use was 25.1 days (SD 15.1), which was 6.7 days (SD 8.8) shorter than the average length of stay in the clinical program (mean 31.8 days, SD 13.3).

Key themes that emerged from provider workgroups were related to the effects of the app development process on engagement and between-session homework completion (eg, “Follow through on homework is better”; “One of the patients I had, he really loved it. He said he felt more accountable. And it involved his wife because he showed his wife so she was kind of onboard with the whole treatment. And he said it felt like [my provider] was in the room...”). Themes emerging from patient workgroups included discussion of personalization of treatment (eg, “I tell [my provider] what kind of topic I want. And then we talk about what times are good. But then she makes up the specifics”), motivation (eg, “Helps the motivation continue”; “It helps remind you on what you need to be doing”), and connection after hours (eg, “You’re [in the IOP clinic] for an allotted amount of time and then you have to be out into the real world… This supplements [the time in the IOP clinic]”). Providers stated that during the final week that patients are in the IOP, providers are focused on discharge planning and therefore less focused on app use by the patients.

**Table 1:** Usability and satisfaction with the Chorus platform and mobile texting apps.

Measure		Score, mean (SD)			
		Patients (n=10)		Providers (n=4)	
**Perceived ease of use**					
	It is simple to use this messaging application.		6.0 (1.5)		5.8 (0.5)
**Perceived usefulness**					
	This messaging application has all the functions and capabilities I expect it to have.		5.7 (1.6)		5.3 (1.0)
	I feel (I am/my patients are) more engaged in my treatment as a result of using this messaging application.		5.8 (1.2)		6.0 (1.4)
**Self-efficacy**					
	I can effectively develop messages with my provider (patient) using this messaging application.		6.0 (1.3)		6.8 (0.5)
	I (my patient) was directly involved in creating the text messages I would receive.		4.8 (2.2)		5.5 (3.0)
	Receiving these text messages is effective in helping me (my patient) complete the treatment plans at home.		5.7 (1.1)		7.0 (0)
**Patient-centeredness**					
	Information provided by the text messages is relevant to me (my patient) and my (patient’s) recovery.		6.2 (1.0)		7.0 (0)
	Overall, I am satisfied with this messaging application.		5.8 (1.4)		5.5 (0.6)

**Illustrative quotes of positive and negative aspects of Chorus.**

**Positive aspects**

- “It's nice to have it remind me of all the things I need to accomplish”
- “Helps the motivation continue”
- “Get out of comfort zone”
- “I feel more encouraged in treatment”
- “It is simple”
- “Very straightforward”
- “Simple to create”
- “Fun texts”
- “Patients love it”
- “Patients like interaction”

**Negative aspects**

- “Harder to use if you're a bad texter”
- “Some bugs”
- “Disappointment it's not a text from a friend”
- “Folders needed [to organize messages]”

## Discussion

This is the first study of a systematic approach for patients and their providers to directly cocreate mobile texting apps for their own use as part of clinical care. We implemented a web-based application development platform (Chorus) and participatory development process in an outpatient clinical setting for patients with severe OCD. Providers required minimal training and were able to cocreate and use apps the same day that they were trained on how to use the platform. Providers and patients determined the content, frequency, and timing of notifications. Despite the high symptom burden experienced by patients, both patients and providers reported that the Chorus platform was easy to use and that it helped patients engage in treatment goals. A total of 9 out of 10 patients agreed that the messages were relevant to them. We observed sustained use of the cocreated apps throughout patients’ time in the IOP. In addition, while the patients and providers were not given specific direction as to the types of messages to create, the 4 categories of messages identified were consistent with key principles of CBT and ERP implemented by the clinic.

There is increasing interest in including patients and providers at various levels of technology development. The Nightscout project is an example of an app first created by a patient’s parent for diabetes management [21]. The parent, who was a computer programmer, identified a need and created a technology to address it, resulting in an open source app for monitoring glucose levels. While this project involved an end user in its development, our approach differs in that computer programming is not required so that apps can be created by individuals without technical backgrounds, including for their own use. Torous and Roux [22] describe an individual patient with schizophrenia working with their provider to create a custom symptom-tracking system. Though the patient and provider were not creating a mobile app, they used spreadsheets to graph symptom counts recorded with a manual tally counter by the patient.

Part of the challenge of involving patients in creating apps for their own use is the high technical and financial barriers to creating apps. The use of application platforms may offer several advantages. Visual development interfaces mean that individuals without knowledge of computer programming can create apps. In addition, compliance, auditing, and security requirements, such as those in the Health Insurance Portability and Accountability Act, can be handled centrally by the platform without the need to repeat these functions and organizational review processes for each individual app. There are several examples of no-code platforms used in health care and research outside of mobile app development. For example, REDCap (Vanderbilt University) [23] and Qualtrics (SAP SE) [24] are commonly used visual development platforms for creating online surveys. Squarespace (Squarespace Inc) [25] and Wix (Wix.com Inc) [26] are platforms to visually create and host websites. This is in addition to platforms for use by developers and institutions with technical expertise, including platforms to facilitate device sensing [27], data collection [28], and data storage and analysis [29].

The direct development of technologies by patients, providers, and other stakeholders has several implications. First, this participatory process aims to increase the level of patient involvement and the relevance of health technologies, which is consistent with recommendations from behavior change theories and guidelines for patient-centered care [7,8,30]. Second, this process also has implications for equity and power sharing, a key principle from participatory approaches such as community-partnered participatory research. If patients are primarily involved at the design stage only (as is the current state of practice), the ability to create and maintain technologies still rests with computer programmers and server infrastructure staff. When patients and providers directly create and maintain their own apps, they may be better able control what is created, change the apps over time as their needs and priorities change, and directly benefit from their own apps.

Our approach is consistent with recent recommendations regarding how health information technologies may be evaluated more effectively within health systems. Learning health systems [31], agile science [32], and responsive research [33] each aim to support iterative intervention development, learning through implementation in real-world settings, and flexible evaluation options such as N-of-1 and pragmatic trials. End users are an untapped talent pool. According to the US census, only 0.1% of the US population are computer programmers [34]. By exploring approaches and technology platforms that do not require programming skills, we are better able to tap into the expertise and capacity of individuals who have lived experiences. An important future consideration is how clinical training programs may be modified to prepare clinicians and other staff for increased patient involvement in health technology development [35].

This study has several limitations. It was conducted at a single site with a limited number of patients, most of whom were white, young (mean 33 years), and had severe OCD symptoms. The clinical program was also suited to texting interventions, given its focus on CBT and exposure-driven treatment. Implementation in larger samples, at other sites, and in other clinical conditions would allow further evaluation of the generalizability of this approach, including how it may be adapted to other technology platforms [11]. This study did not evaluate the impact of the app development and cocreation process on clinical outcomes, a topic to be explored in randomized controlled trials. While patients engaged with the app for the majority of their length of stay in the IOP, we did observe a discontinuation of the app an average 6.7 days prior to discharge from the IOP. This may be due to the reduced focus on exposures and interventions in the final week of the IOP, with a transition to discharge planning reported by providers.

Technology approaches that use flexible, user-driven platforms to engage a broader set of individuals in development may hold potential for increasing the relevance and sustainability of technology interventions, which may in turn lead to improved patient engagement and outcomes.

## Appendix

Multimedia Appendix 1 Patient Usability Measure.

## Abbreviations

CBT: cognitive behavioral therapy
ERP: Exposure and Response Prevention
IOP: intensive outpatient program
OCD: obsessive-compulsive disorder
UCLA: University of California Los Angeles
Y-BOCS: Yale-Brown Obsessive-Compulsive Scale
