# Supplementary material for: Patient and Provider Cocreation of Mobile Texting Apps to Support Behavioral Health: Usability Study
Source: JMIR Mhealth Uhealth. 2020 Jul 29;8(7):e12655. doi: 10.2196/12655 (PMC7424494; doi:10.2196/12655)
Supplement: Multimedia Appendix 1 [file mhealth_v8i7e12655_app1.pdf]

Name: ______________________________ Date: ______________

Chorus Messaging Application Study - Patient Survey

	Strongly Disagree			Neutral			Strongly Agree	
1. It is simple to use this messaging application.	1	2	3	4	5	6	7	NA
2. I feel comfortable using this messaging application.	1	2	3	4	5	6	7	NA
3. It was easy to learn to use this messaging application.	1	2	3	4	5	6	7	NA
4. I believe I became productive quickly using this messaging application.	1	2	3	4	5	6	7	NA
5. Whenever I make a mistake using the messaging application, I recover easily and quickly.	1	2	3	4	5	6	7	NA
6. The web interface of this messaging application is pleasant.	1	2	3	4	5	6	7	NA
7. I like using the web interface of this messaging application.	1	2	3	4	5	6	7	NA
8. This messaging application has all the functions and capabilities I expect it to have.	1	2	3	4	5	6	7	NA
9. I can effectively develop messages with my provider using this messaging application.	1	2	3	4	5	6	7	NA
10. I feel more engaged in my treatment as a result of using this messaging application.	1	2	3	4	5	6	7	NA
11. Overall, I am satisfied with this messaging application.	1	2	3	4	5	6	7	NA
12. I was directly involved in creating the text messages I would receive.	1	2	3	4	5	6	7	NA
13. Information provided by the text messages is relevant to me and my recovery.	1	2	3	4	5	6	7	NA
14. The information provided by the text messages is easy to understand.	1	2	3	4	5	6	7	NA
15. Receiving these text messages is effective in helping me complete the treatment plans at home.	1	2	3	4	5	6	7	NA
16. I feel comfortable interacting with these text messages.	1	2	3	4	5	6	7	NA

List the most **negative** aspect(s):

1. ______________________________
2. ______________________________
3. ______________________________

List the most **positive** aspect(s):

1. ______________________________
2. ______________________________
3. ______________________________
